# Quinones as Key Intermediates in Natural Products Synthesis. Syntheses of Bioactive Xanthones from *Hypericum perforatum*

**DOI:** 10.3390/molecules14082857

**Published:** 2009-08-03

**Authors:** George A. Kraus, John Mengwasser

**Affiliations:** Department of Chemistry, Iowa State University, Ames, Iowa 50011, USA; E-mail: jhm2876@iastate.edu (J.M.)

**Keywords:** photoacylation, green chemistry, xanthones

## Abstract

Two bioactive xanthones from *Hypericum perforatum* have been synthesized by direct routes. Benzo[c]xanthone **5** can be prepared from intermediate **4**.

## Introduction

Because of their extensive use as botanical dietary supplements, there is increasing interest in the chemical constituents of *Hypericum* and *Echinacea* [1]. Although some components of *Hypericum* species such as hypericin and hyperforin are well known, these species contain many additional bioactive components, including flavones, procyanidins and xanthones [2]. Among the many xanthones found in *Hypericum* species, euxanthone (**1**) and 1-methoxy-7-hydroxyxanthone (**2**) are found in *Hypericum perforatum* [3]. 

**Figure 1:**
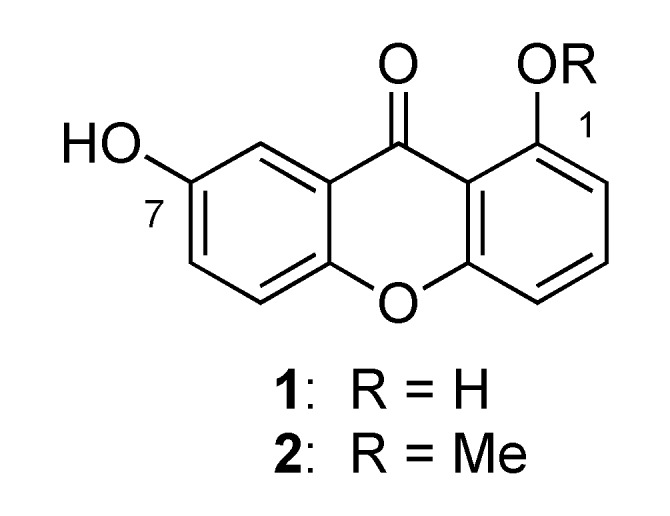
Xanthones from Hypericum perforatum.

Euxanthone exhibits a range of potentially useful biological activities. Recently, researchers reported that euxanthone promotes neurite outgrowth by selectively activating the MAP kinase pathway [4]. Euxanthone showed inhibitory effects on the growth of *Plasmodium falciparum* with IC_50_ values in the milligram/milliliter level [5]. Euxanthone also inhibited HIV-1 reverse transcriptase with IC_50_ values at the milligram/milliliter level [6]. In the vasodilatation assay, both xanthones **1** and **2** exhibited relaxing activity on the contractions evoked by potassium chloride in rat thoracic aorta rings in a dose-dependent manner [7]. Euxanthone has been synthesized by heating hydroquinone and ethyl 2,6-dihydroxybenzoate in boiling diphenyl ether to make **1** [8]. Our synthetic route to **1** requires three steps from two commercially available starting materials and is amenable to scale up. See Figure 1. 

## Results and Discussion

As shown below, the key step in our synthetic route involves a photoacylation [9] using 2,6-dimethoxybenzaldehyde and benzoquinone. We developed the photoacylation of quinones as a green alternative to certain Friedel-Crafts reactions. This reaction, which is a nice example of atom economy, produces adducts which have been used in syntheses of benzodiazepines and natural products such as frenolicin [9]. Before the synthesis of **3**, we had not demonstrated this reaction with hindered aldehydes. Since this reaction occurs via an acyl radical, we were concerned that an intramolecular radical translocation to the methyl of the methoxyl group might intervene before the desired intermolecular reaction of the acyl radical with the quinone. 

**Scheme 1:**
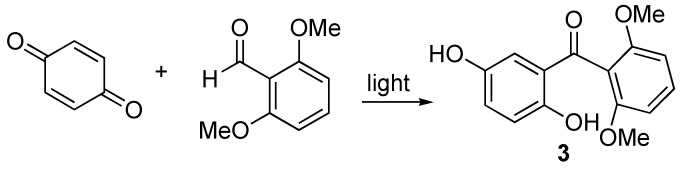
Photoacylation reaction.

To show the scope of this reaction we synthesized adduct **4** in 76% yield from naphthoquinone. Adduct **4** could be converted into xanthone **5** in 61% yield by demethylation with boron tribromide, followed by heating at 180 °C for 16 hours. 

**Scheme 2:**
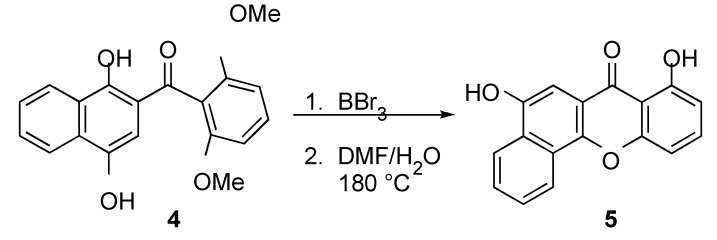
Cyclization to xanthone **5**.

To synthesize xanthone **2**, we treated adduct **3** with potassium hydroxide in boiling methanol for 12 hours [10]. Demethylation of **2** using boron tribromide afforded euxanthone (**1**) in 56% yield as yellow crystals. 

**Scheme 3:**
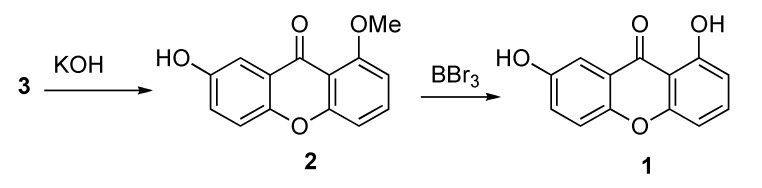
Synthesisof **1** and **2**.

In summary, two bioactive components from *Hypericum perforatum* have been synthesized by direct routes. These results further extend the synthetic utility of the photoacylation of quinones. 

## Experimental

### General

Unless stated otherwise, all reactions were magnetically stirred and monitored by thin-layer chromatography (TLC) using 0.25 mm precoated silica gel F254 plates (Sigma-Aldrich). Column or flash chromatography was performed with the indicated solvents using silica gel (230-400 mesh) purchased from Dynamic Adsorbents, LLC. All melting points were obtained on a Laboratory Devices capillary melting point apparatus and are uncorrected. ^1^H- and ^13^C-NMR spectra were recorded in CDCl_3_ on a Bruker VXR-400 (400/100 MHz) spectrometer. Chemical shifts are reported relative to internal chloroform (^1^H, 7.26 ppm; ^13^C, 77.23 ppm). High resolution mass spectra were performed at the Iowa State University Mass Spectrometry Laboratory.

### Representative Photochemical Procedure

*(1,4-Dihydroxynaphthalen-2-yl)(2,6-dimethoxyphenyl)-methanone* (**4**): Naphthoquinone (98 mg, 0.62 mmol), 2,6-dimethoxybenzaldehyde (95 mg, 0.565 mmol) and benzophenone (31 mg, 0.17 mmol) were dissolved in dry benzene (19 mL) and degassed with argon for 15 min. The solution was then irradiated for 5 days with a high-pressure Hg-vapor lamp equipped with a Pyrex filter. The solution was concentrated *in vacuo* and the residue was purified by column chromatography (hexanes-EtOAc 19:1 1:3) to afford 139 mg (76%) of **4** as bright yellow needles, m.p. 194-196 °C; ^1^H-NMR (CDCl_3_): δ 13.31 (s, 1H), 8.52 (d, *J* = 8.00 Hz, 1H), 8.08 (d, *J* = 8.00 Hz, 1H), 7.68 (t, *J* = 8.00 Hz, 1H), 7.59 (t, *J* = 8.00 Hz, 1H), 7.38 (t, *J* = 8.00 Hz, 1H), 6.64 (d, *J* = 8.00 Hz, 2H), 6.54 (s, 1H), 4.84 (s, 1H), 3.74 (s, 6H); ^13^C-NMR (CDCl_3_): δ 200.4, 157.8, 157.4, 143.0, 131.4, 129.9, 129.8, 126.1, 124.8, 121.8, 116.8, 113.9, 107.7, 104.2, 56.2; HRMS (EI) m/z calcd. for C_19_H_16_O_5_ 324.0998, found 324.0998.

*(2,5-Dihydroxyphenyl)(2,6-dimethoxyphenyl)methanone* (**3**): In the reaction between benzoquinone and 2,6-dimethoxybenzaldehyde, the product, **3**, was inseparable from the aldehyde starting material; ^1^H- NMR (acetone-d_6_) δ 11.71 (s, 1H), 7.46 (t, *J* = 8.00 Hz, 1H), 7.09 (dd, *J* = 8.00 Hz, *J* = 4.00 Hz, 1H), 6.87 (d, *J* = 8.00 Hz, 1H), 6.80 (d, *J* = 8.00 Hz, 2H), 6.70 (d, *J* = 4.00 Hz, 1H), 3.73 (s, 6H); HRMS (EI) m/z calcd. for C_15_H_14_O_5_ 274.0841, found 274.0846.

*5,8-Dihydroxy-7H-benzo[c]xanthen-7-one* (**5**): An oven-dried 50-mL round bottom flask equipped with a stir bar was charged at room temperature (rt) with benzophenone **4** (27.3 mg, 0.08417 mmol, 1 equiv), followed by CH_2_Cl_2_ (3.0 mL), giving a bright yellow solution. Then BBr_3_ (337 μL, 1.0 M in CH_2_Cl_2_, 0.337 mmol, 4 equiv) was added via syringe. This instantly generated a deep red, homogeneous solution. After 8 h at rt, the reaction mixture was cooled to 0 °C and then H_2_O (2 mL) was added via syringe, resulting in an orange biphasic mixture. This was warmed to rt, diluted with CH_2_Cl_2_ (20 mL), poured into a separatory funnel and then the organic layer was washed with H_2_O (2 x 20 mL), brine (1 x 20 mL), dried over MgSO_4_, filtered and concentrated *in vacuo* to afford an orange solid that was purified by column chromatography (hexanes-EtOAc 3:2) to afford the tetrahydroxybenzophenone as an orange solid (16 mg, 0.0540 mmol, 64%); ^1^H-NMR (acetone-d_6_): δ 8.67 (s, 1H, exchangeable with D_2_O), 8.57 (s, 1H, exchangeable with D_2_O), 8.44 (d, *J* = 8.0 Hz, 1H), 8.20 (d, *J* = 8.0 Hz, 1H), 7.71 (t, *J* = 8.0 Hz, 1 H), 7.62 (t, *J* = 8.0 Hz, 1 H), 7.18 (t, *J* = 8.0 Hz, 1 H), 6.78 (s, 1 H), 6.55 (d, *J* = 8.0 Hz, 2 H); ^13^C-NMR (acetone-d_6_): δ 202.3, 156.3, 145.5, 132.0, 130.9, 130.2, 127.1, 126.7 (overlap of two C), 124.8, 123.1, 115.9, 115.4, 108.1, 107.9.

A pressure tube equipped with a stir bar was charged with the tetrahydroxybenzophenone (18.9 mg, 0.0638 mmol), H_2_O (1.6 mL) and DMF (1.0 mL). The resulting homogeneous orange solution was cooled to 0 °C, sealed and then warmed to 180 °C. After 16 h at this temperature, a yellow solid precipitated. The mixture was cooled to 0 °C, opened, diluted with EtOAc (10 mL), and poured into a separatory funnel. The organic layer was washed with H_2_O (2 x 10 mL) and brine (1 x 10 mL), dried over MgSO_4_, filtered and concentrated *in vacuo* to afford a yellow solid that was purified by column chromatography (hexanes-EtOAc 9:1) to afford the xanthone **5** as a yellow solid (17 mg, 0.0638 mmol, 95% yield); **^1^**H-NMR (DMSO-d_6_): δ 12.72 (s, 1 H, exchangeable with D_2_O), 8.60 (d, *J* = 8.0 Hz, 1 H), 8.27 (d, *J* = 8.0 Hz, IH), 7.81 (dt, *J* = 8.0 Hz, 2H), 7.73 (t, *J* = 8.0 Hz, IH), 7.34 (s, 1 H), 7.26 (d, *J* = 8.0 Hz, IH) 6.82 (d, *J* = 8.0 Hz, IH); ^13^C-NMR (DMSO-d_6_) δ 181.5, 161.0, 155.8, 150.5, 147.8, 137.0, 130.2, 129.5, 128.4, 124.6, 123.3, 123.1, 116.8, 110.4, 109.0, 107.9, 98.8. 

*1-Methoxy-7-hydroxyxanthone* (**2**): To a mixture (87.2 mg) of **3** and inseparable 2,6-dimethoxy-benzaldehye in ice-cold methanol (2 mL), a solution of KOH (89.2 mg, 1.6 mmol) in methanol (1 mL) was added dropwise over 2 min. The reaction mixture was refluxed overnight, cooled to 0 °C, then acidified with 10% HCl. After extracting with EtOAc (2x), the combined organic layers were dried over MgSO_4_, filtered, concentrated *in vacuo*, and purified by column chromatography (3% MeOH in CH_2_Cl_2_) to afford 43 mg of **2** that crystallized from MeOH as yellow needles, m.p. 238-240 °C (lit. [11] 238-240 °C). All spectra were identical with those previously reported [11].

*Euxanthone* (**1**): To a stirred suspension of **2** (48 mg, 0.198 mmol) in CH_2_Cl_2_ (2 mL) at –78 °C was rapidly added BBr_3_ (149 mg, 0.595 mmol, 0.595 mL of 1.0 M solution in CH_2_Cl_2_). The reaction mixture was slowly allowed to warm to rt, stirred for 36 hours, cooled to 0 °C, then quenched with water. CH_2_Cl_2_ was removed *in vacuo*, and the aqueous layer was extracted with EtOAc (2x). The combined organic layers were washed with water and brine and dried over MgSO_4_, filtered, concentrated *in vacuo*, and purified by column chromatography (15% EtOAc in hexanes) to afford 23.5 mg (56% yield) of **1** as yellow needles, m.p. 236-238 °C (lit. [12] 236-238 °C).
